# Molecular Mobility in Hyperbranched Polymers and Their Interaction with an Epoxy Matrix

**DOI:** 10.3390/ma9030192

**Published:** 2016-03-15

**Authors:** Frida Román, Pere Colomer, Yolanda Calventus, John M. Hutchinson

**Affiliations:** Departament de Màquines i Motors Tèrmics, ESEIAAT, Universitat Politècnica de Catalunya, Terrassa 08222, Barcelona, Spain; roman@mmt.upc.edu (F.R.); colomer@mmt.upc.edu (P.C.); calventus@mmt.upc.edu (Y.C.)

**Keywords:** dielectric relaxation spectroscopy (DRS), differential scanning calorimetry (DSC), dynamic mechanical analysis (DMA), hyperbranched polymer, epoxy, poly(ethyleneimine)

## Abstract

The molecular mobility related to the glass transition and secondary relaxations in a hyperbranched polyethyleneimine, HBPEI, and its relaxation behaviour when incorporated into an epoxy resin matrix are investigated by dielectric relaxation spectroscopy (DRS) and dynamic mechanical analysis (DMA). Three systems are analysed: HBPEI, epoxy and an epoxy/HBPEI mixture, denoted ELP. The DRS behaviour is monitored in the ELP system in three stages: prior to curing, during curing, and in the fully cured system. In the stage prior to curing, DRS measurements show three dipolar relaxations: γ, β and α, for all systems (HBPEI, epoxy and ELP). The α-relaxation for the ELP system deviates significantly from that for HBPEI, but superposes on that for the epoxy resin. The fully cured thermoset displays both β- and α-relaxations. In DMA measurements, both α- and β-relaxations are observed in all systems and in both the uncured and fully cured systems, similar to the behaviour identified by DRS.

## 1. Introduction

Cured epoxy resins are thermosets exhibiting a crosslinked structure with excellent electrical properties, good chemical and corrosion resistance, low shrinkage, and high tensile strength and modulus. As a consequence of characteristics such as these, epoxies are widely used in applications such as coatings, adhesives and electronic encapsulation. The wide range of curing agents available for their crosslinking makes them extremely versatile. However, inherent brittleness can limit their use in certain circumstances. To obtain an acceptable set of properties they must be converted from oligomers or monomers of low molecular weight into a highly crosslinked network polymer by means of curing agents in which the crosslinking reaction can occur via a variety of reaction mechanisms [1,2,3].

Recently, the use of dendrimers and hyperbranched polymers as curing agents has come to some prominence. Dendrimers are molecules with a three-dimensional globular shape [4,5,6], in which well-defined highly branched molecules emanate from a central core and have a large number of functional groups at the periphery. This molecular architecture endows dendrimers with unusual chemical and physical properties, such as their extraordinarily high functionalities [7], and has led to their use in diverse applications. For example, poly(propylene imine) dendrimers have been shown to plasticize a thermoplastic, polyvinyl chloride [8], the plasticization effect being considered to occur as a result of additional free volume from the highly branched structure of the dendrimer. However, it is their use as curing agents for thermosets, quite widely reported in the literature recently, which is of particular interest here. Some authors [9,10] have used polyamido-amine dendrimers grafted onto silica as the curing agent in epoxy systems, and report increased thermal stability in comparison with the same epoxy cured with ethylene diamine (EDA), but there was no consensus on whether the glass transition temperature is increased or not. More generally, the reaction kinetics of bisphenol-A epoxy resins cured with polyamido-amine dendrimers has been studied [11,12], and it has been reported that, in comparison with the use of EDA as curing agent, the curing kinetics using polyester-amine dendrimers is very different, presenting two stages of cure and less heat of reaction [13]. The use of polypropylene-imine dendrimers as curing agents in epoxy systems is quite widely reported [14,15,16], and it appears that the molecular architecture of the dendrimers, in particular their steric hindrance and the number of attached –OH groups [14], greatly affects the reaction kinetics. It is clear that suitable dendrimers can effectively crosslink epoxy resins, and this may be advantageous if the dendrimers exhibit improved compatibility with epoxy resins over the conventional linear aliphatic amine curing agents of low molecular weights. Nevertheless, it is also clear that the reaction kinetics can be strongly influenced by the nature of the dendrimer, and that this is an area for investigation.

Hyperbranched polymers (HBP) are a type of dendronised polymers which can be used as effective polymer modifiers of thermosetting materials as a consequence of their high degree of branching and their high concentration of surface reactive groups, which can become covalently linked to the epoxy matrix or can be modified in order to enhance their compatibility with the epoxy resin. New developments and applications based upon the use of HBP are numerous [17,18], and the reaction kinetics of a wide variety of epoxy systems with HBP has been studied, including tetrafunctional epoxy systems [19], anhydride cured [20] and cationically intitated epoxy systems [21], and UV-cured epoxy systems for which significant improvements in toughness were reported [22,23]. Recently, the use of Lupasol^®^, a hyperbranched poly(ethyleneimine) (HBPEI) produced by BASF, as a multifunctional crosslinker of epoxy resins has been reported. HBPEI can react with epoxy monomers by an epoxy-amine condensation mechanism and is thus incorporated into the network structure of the thermoset [24]. The densely branched architecture of high molecular weight Lupasol^®^ creates significant mobility restrictions, which can have a strong effect on the reaction kinetics, and hence on the glass transition temperature and on the degree of crosslinking of the cured material [25]. For example, a comparison of HBPEI and diethylene triamine (DETA) as curing agents for diglycidyl ether of bisphenol-A (DGEBA) epoxy resin showed not only that the cure kinetics with the HBP were slightly delayed in comparison with DETA, but also that gelation occurs earlier and vitrification occurs later for HBPEI in comparison with DETA, and that the glass transition temperature of the fully cured system, *T*_g∞_, is higher when DETA is used, which was attributed to the greater network stiffness associated with the amount of aromatic rings in the network structure [24]. Another illustration of the significant changes that can occur in the cure kinetics when HBPEI is used as curing agent instead of an aliphatic low molecular weight amine may be found in the field of polymer layered silicate nanocomposites: when a DGEBA-clay mixture is cured with a polyoxypropylene diamine, the degree of exfoliation is poor as a consequence of too rapid a cure of the bulk extra-gallery epoxy, whereas when the cure is effected with HBPEI the extra-gallery reaction rate is slower, allowing more time for the intra-gallery reaction to occur and resulting in a significant improvement in the degree of exfoliation in the cured nanocomposite [26].

A better understanding of the interaction between the HBP and the epoxy resin during cure, and hence of the effect of the reaction kinetics on the final network structure, may be obtained from dielectric relaxation spectroscopy (DRS), which provides information about the segmental mobility within a polymer. Such studies can be applied to both the epoxy resin and the HBP separately, as well as to their mixture and to the system during cure and in the fully cured state. There is a large body of literature devoted to the study of the evolution of the primary and secondary relaxations in various epoxy resins, in large part DGEBA, and of epoxy-based mixtures which form networks by a condensation reaction, principally with diamines. For the DGEBA epoxy alone, for example, Casalini *et al.* [27] and Capaccioli *et al.* [28] report a glass transition (α-relaxation) and only one secondary relaxation, denoted as the γ-relaxation, though later they report two secondary relaxations, β and γ [29], as has also been reported by others [30] as well as ourselves [31]. The observation by Gallone *et al.* [32] of only one secondary relaxation in neat DGEBA resin at 25 °C is because this temperature is above the cross-over between the α- and β-relaxations.

In respect of the dielectric analysis of the curing epoxy-diamine system, a good starting point is the excellent review of Senturia and Sheppard [33]. In some early work of Mangion and Johari [34,35,36] where the effect of the extent of isothermal cure was investigated, there are clearly two secondary relaxations in the reacting mixture in addition to the glass transition, and these relaxations are also clearly evident in non-isothermal DRS of the epoxy-amine system [31]. The changes that take place during cure in each of these secondary relaxations, as well as in the α-relaxation, have been the subject of much discussion, from both experimental (e.g., [32,34,35,36,37,38,39,40,41]) and theoretical standpoints (e.g., [42,43,44,45]), including, in particular, interpretation of vitrification during isothermal cure [38,39,46,47].

On the basis of such studies, it is possible to assign certain molecular motions to each of the secondary relaxations in these epoxy systems. For example, the strength of the γ-relaxation decreases with cure [34,48], and it is not observed in the fully cured system [31], and thus it is generally agreed that the γ-relaxation in the pure epoxy and in the epoxy-amine system is related with the dynamics of dipoles localized in the epoxide end groups [30], or more generally with the local motions of dipoles of unreacted species [34]. As regards the β-relaxation, the early studies of Ochi and co-workers [49] by dynamic mechanical analysis (DMA) of anhydride cured epoxy systems showed that it was affected by the chemical structure of the curing agents but not by that of the epoxy resins, and subsequently from combined dielectric analysis (DEA) and DMA they assigned the β-relaxation to the formation of H-bonds between hydroxyl ether groups and methoxy branches [50]. Later, Mangion and Johari [34] observed by DEA that the β-relaxation increases during cure, and assigned it to the local motion of dipolar groups that are created during the cross-linking reaction, particularly hydroxyl-ether groups but also secondary and tertiary amines.

The molecular dynamics of a wide variety of dendrimers [51,52,53,54] and, in particular, HBPs, including hyperbranched polyurethanes [55,56], polyglycerols [57,58], polyesters [59,60,61], and polyamide systems [62,63], have been studied by dielectric spectroscopy. In most cases, these dendrimers and HBPs display a glass transition and two secondary relaxations, the γ-relaxation invariably being attributed to motion of the terminal groups. In contrast, there are rather fewer studies of the molecular dynamics of cross-linking systems in which HBPs are incorporated, and of how the relaxation behaviour changes as the cross-linking reaction proceeds. Boiteux and co-workers [64,65,66] investigated the molecular dynamics in polyurethane networks cross-linked with hyperbranched polyesters and observed that the secondary β- and γ-relaxations were unaffected by the degree of cross-linking of the network, which was modified by varying the polyurethane chain length. On the other hand, Maroulas *et al.* [67] investigated the molecular dynamics in hyperbranched polyimides cross-linked with ethylene glycol diglycidyl ether and found that the γ-relaxation, which was faster in the hyperbranched system than in its linear analogue, becomes slower but increases in magnitude as the cross-linking proceeds. This apparently anomalous behaviour was attributed to the sum of two opposing effects: an increase in free volume accompanied by increased constraint as a result of the crosslinking. The influence of free volume on the γ-relaxation was also suggested by the work of Zhu *et al.* [61], who found that the γ-relaxation temperature increased with generation number in hyperbranched polyester systems. This was interpreted in terms of a coupling of the molecular motions of the terminal groups with the α-relaxation glass transition, in other words that the γ-relaxation was influenced by its environment.

**Figure 1:**
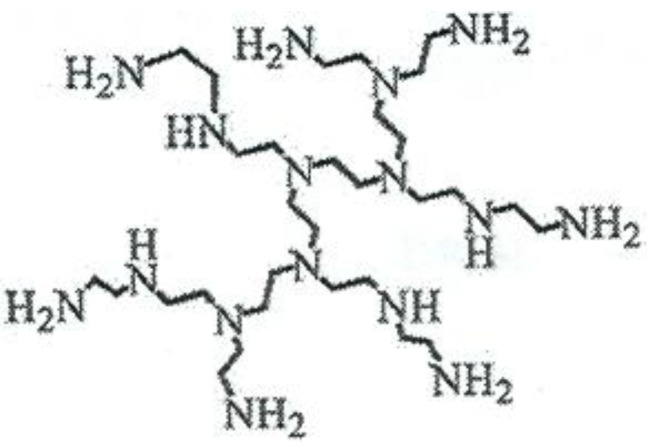
Schematic illustration of the hyperbranched polyethyleneimine, HBPEI.

It is our belief that the interactions between the HBP and the matrix, such as those discussed above, have an important effect on the reaction kinetics of the curing system. In particular, our own observation [26,68] that an improved nanostructure is developed in an epoxy-clay nanocomposite when cured with HBPEI rather than a polyoxypropylene diamine must be related to the molecular dynamics interactions between the HBPEI and the epoxy. As part of a wider study in which the effects of the molecular weight of the HBPEI and of the molecular architecture of the HBP are currently being investigated, we present here the results of a molecular dynamic study of the following: the pure HBPEI (trade name Lupasol PR8515, BASF), shown schematically in Figure 1; the neat epoxy resin (diglycidyl ether of bisphenol-A, DGEBA); the unreacted mixture of HBPEI and epoxy, denoted ELP; the ELP system during non-isothermal cure; and the fully cured system.

## 2. Results

### 2.1. Optical Microscopy

**Figure 2:**
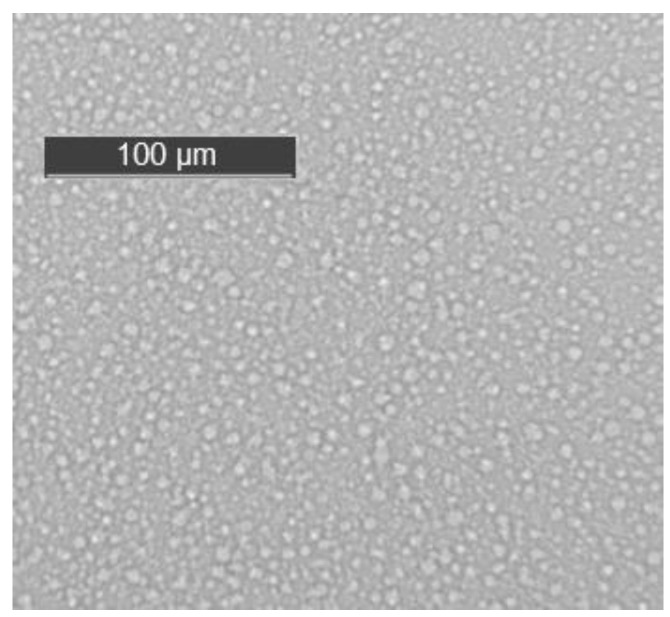
Morphology of the uncured ELP system.

Preliminary studies by transmission optical microscopy showed that the morphology of the uncured mixture of epoxy and HBPEI (ELP sample), after degassing, consisted of two phases at room temperature, as seen in Figure 2. The HBPEI appears as globules of the order of 5 μm diameter or less within the epoxy matrix.

### 2.2. Thermogravimetric Analysis (TGA) and Differential Scanning Calorimetry (DSC) Results

**Figure 3:**
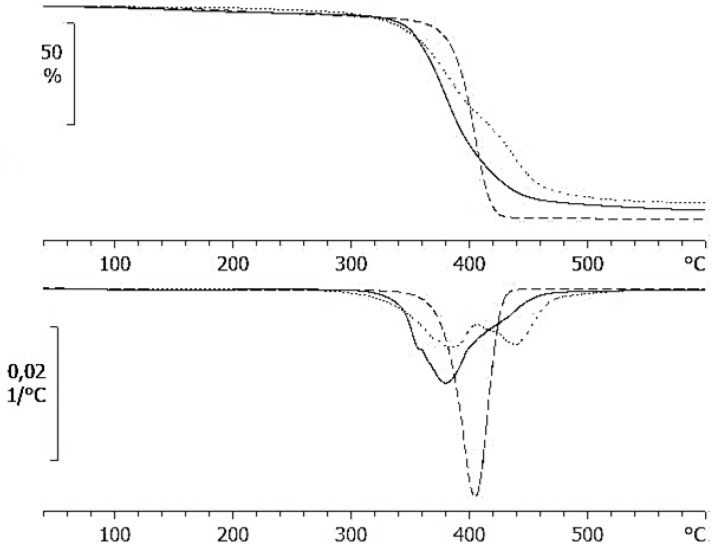
Comparative thermogravimetric results for the epoxy (dotted line), HBPEI (dashed line) and ELP (full line), for a heating rate of 10 K/min: upper graph, % weight loss as a function of temperature; lower graph, differential thermogravimetric analysis (DTGA) curves.

Thermogravimetric analysis (TGA) carried out at different heating rates over the temperature range from 40 to 600 °C shows that the thermal degradation of the HBPEI sample occurs in a single stage, while the epoxy degradation occurs in two clearly defined stages, as seen in Figure 3. The ELP sample shows a behaviour intermediate between these two, as if there are two overlapping processes. The two-stage degradation of epoxy has been observed previously [69,70,71]: the first stage was attributed to isomerization of the epoxy groups and subsequent C–O bond scission, liberating acrolein, allyl alcohol and acetone as the principal volatile products, with homopolymerization of the epoxy resin then reducing the rate of weight loss before the second stage of degradation.

Table 1 gives the characteristic temperatures of the degradation processes of the three samples studied here, corresponding to: 5% weight loss, *T*_5%_; the onset temperature, *T*_onset_, defined as the intersection of the initial slope with the inflectional tangent; the DTGA peak temperature, *T*_DTGA_. These results show that the onset temperature for the HBPEI is significantly greater than those for the other two samples, even though the first 5 wt % is lost more quickly for the HBPEI.

**Figure 4:**
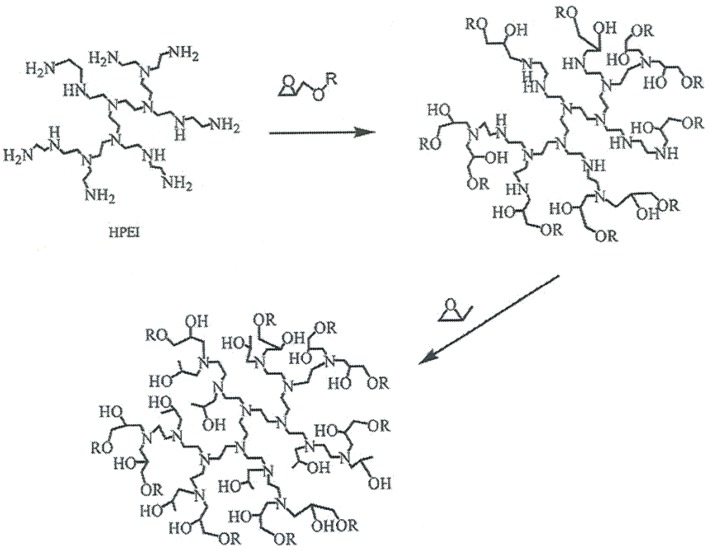
Schematic illustration of the curing reaction of the epoxy resin with HBPEI.

The non-isothermal curing reactions, for which the reaction scheme is illustrated in Figure 4, were carried out in a differential scanning calorimeter (DSC), under the conditions given in the Experimental section. From the heat flow curve, the glass transition temperature of the uncured system, *T*_g0_, was determined and the exothermic cross-linking reaction was obtained. In the immediately subsequent second scan, the glass transition temperature of the fully cured sample, *T*_g∞_, was determined.

**Figure 5:**
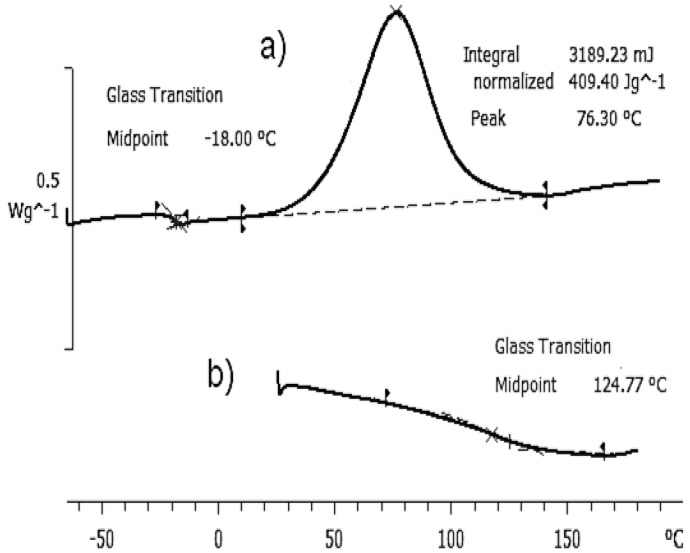
Thermogram of ELP sample: (**a**) first scan, at 2 K/min; (**b**) second scan, at 10 K/min. The exothermic direction is upward.

Figure 5 shows the thermogram of the cross-linking reaction of the ELP mixture at a heating rate of 2 K/min. The upper diagram, Figure 5a, shows a single glass transition at a temperature *T*_g0_ below 0 °C, and an exothermic peak between approximately 10 °C and 140 °C corresponding to the curing reaction, this peak being characterised by the peak temperature, *T*_p_, and the area, shown in Figure 5a, which corresponds to the heat of reaction, Δ*H*. In the second scan, shown in the lower diagram, Figure 5b, it can be seen that there is no residual cure reaction, implying that the sample was fully cured after the previous non-isothermal cure, and the rather broad glass transition of the fully cured sample is observed between 100 and 150 °C, the *T*_g∞_ being determined as the mid-point temperature between the glassy and rubbery asymptotes, as shown in Figure 5b.

Table 2 gives the summary of the DSC results for the three samples studied at the different heating rates: 2 and 10 K/min for the pure components HBPEI and DGEBA, from 0.5 to 10 K/min for the ELP curing reaction, and 10 K/min for the cured ELP plaque. It can be seen that, for a heating rate of 2 K/min, the glass transition temperature for the HBPEI, −55.4 °C, is significantly lower than that for the epoxy resin, −17.2 °C, while the ELP mixture displays a single glass transition at −18.0 °C. The appearance of a single *T*_g_ in the mixture would suggest that it is a miscible blend, contrary to the observation of a heterogeneous mixture observed by optical microscopy and shown in Figure 2. There are several expressions for calculating the *T*_g_ of a soluble blend of two polymers [72,73,74,75], but perhaps the most widely used is the so-called Fox equation [73].

1/*T*_g_ = *w*_1_/*T*_g1_ + *w*_2_/*T*_g2_ (1)

where w_1_ and w_2_ are the weight fractions of the two components, and *T*_g1_ and *T*_g2_ are their glass transition temperatures, respectively. If Equation 1 is applied in the present case, we obtain *T*_g_ = −24.4 °C for the ELP mixture, somewhat different from the experimental value of −18.0 °C, which is instead very much closer to the *T*_g_ of the pure epoxy. It seems therefore that by DSC we determine, for this ELP mixture, a glass transition temperature that reflects essentially that of the major component, *i.e.*, the epoxy resin, albeit slightly depressed, perhaps by the effect of an increase in free volume on being mixed with the HBPEI, while there is no detectable transition for the HBPEI phase in the mixture. On the other hand, as will be seen in a later section, the measurements made by dielectric relaxation spectroscopy (DRS) show two alpha relaxations for the ELP mixture, one characteristic of the epoxy resin and the other characteristic of the HBPEI.

The results collected in Table 2 further show that the heat of reaction is approximately 400 J/g, or about 90 kJ per epoxy equivalent (ee). This value is rather lower than that of the epoxy-diamine reaction, in the range 104 to 108 kJ/ee, but is close to the value obtained for the same epoxy resin when cured non-isothermally, at relatively high heating rates, with a polyoxypropylene diamine in the presence of organically modified clay in layered silicate nanocomposite systems [76]. In the case of the nanocomposite systems, the heat of reaction decreased at fast heating rates as a consequence of the occurrence of an epoxy homopolymerization reaction, catalyzed by the onium ion of the clay, the homopolymerization reaction having a lower heat of reaction, of the order of 92 kJ/ee [77,78]. We believe the same effect is occurring here, with some epoxy homopolymerization being catalyzed by the numerous −OH groups produced during the reaction (see scheme, Figure 4).

It is also possible to determine the activation energy for the curing reaction, using the Kissinger method [79,80]. This method assumes that the degree of cure at the peak temperature, *T*_p_, is the same for all heating rates, β, and the activation energy is then determined from the slope of a plot of ln(β/*T*_p_^2^) *versus* 1/*T*_p_. The results for *T*_p_ listed in Table 2 give a value of 63.3 kJ/mol for the curing reaction of the ELP system, which is in good agreement with the value that can be found from the cure of the same epoxy resin with the same HBPEI in nanocomposites systems, for the sample with no clay content and over a higher heating rate range from 10 to 20 K/min [81]. Interestingly, this value is slightly higher than the values obtained for the non-isothermal cure of the same epoxy resin in nanocomposite systems cured with a polyoxypropylene diamine, which were in the range 49 to 54 kJ/mol [76].

Finally, the glass transition temperature of the fully cured sample, obtained from the second scan after cure at the four different rates shown in Table 2, has an average value of *T*_g∞_ = 123.5 ± 2.0 °C. This compares very well with the value obtained for the cured plaque, which was isothermally cured and post-cured and then scanned at 10 K/min in the DSC.

### 2.3. Dielectric Relaxation Spectroscopy (DRS) Results

#### 2.3.1. Hyperbranched Polyethyleneimine (HBPEI)

**Figure 6:**
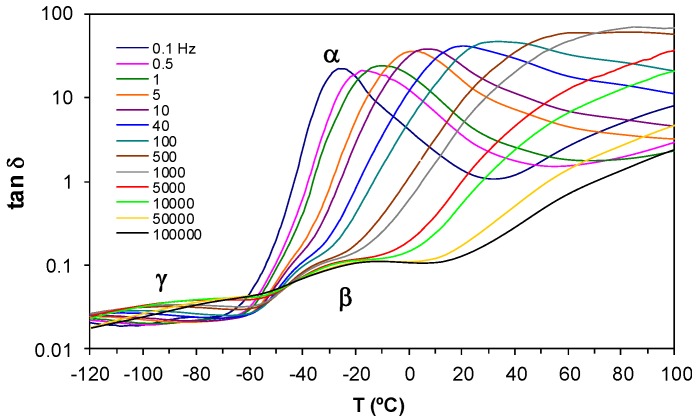
Dielectric relaxation spectroscopy (DRS) spectra for the HBPEI at a heating rate of 0.5 K/min.

The dielectric spectra for the HBPEI obtained at a heating rate of 0.5 K/min and over a frequency range from 0.1 Hz to 100 kHz are shown in Figure 6. This Figure shows that there are two secondary relaxations in this hyperbranched polymer. The γ-relaxation appears at temperatures lower than about −60 °C, and is attributed to the movements of the end groups (NH_2_) of the HBPEI. At temperatures between about −60 °C and +10 °C, and particularly at higher frequencies, the β-relaxation appears, which is attributed to the molecular mobility of segments within the branches. The α-relaxation, of much greater intensity than either of the secondary relaxations, is associated with the glass transition of the hyperbranched polymer.

**Figure 7:**
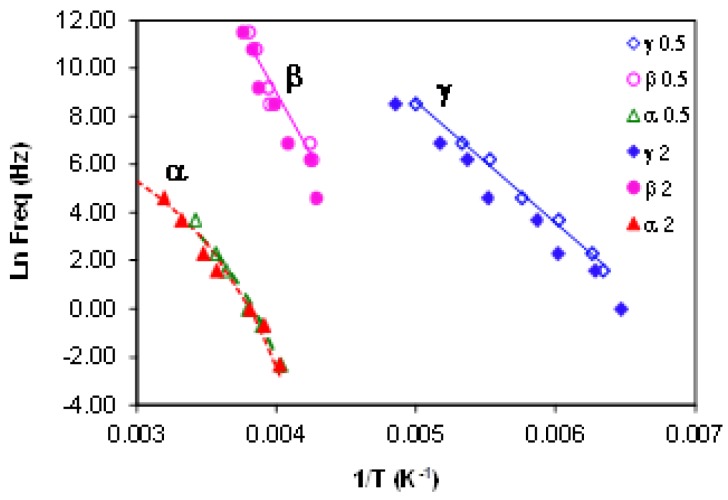
Relaxation map for HBPEI at two different heating rates: 0.5 K/min (open symbols) and 2 K/min (filled symbols).

From the frequency dependence of the peak temperature for each of these three relaxations, the relaxation map for the neat HBPEI can be constructed, and is shown in Figure 7. An Arrhenius dependence on temperature can be seen for the β- and γ-relaxations, while the α-relaxation shows a slight curvature which is characteristic of the Vogel-Fulcher-Tammann (VFT) equation [82]. The β- and γ-relaxations are independent from the α-relaxation; in particular, the β-relaxation does not present the appearance of a Johari-Goldstein (JG) relaxation, according to its common classification [83], in contrast to what is observed for the pure epoxy resin, as is shown immediately below. For some other hyperbranched polymers, such as polyglycerols [57] and polyester amides [84], the β-relaxation presents a Johari-Goldstein behaviour, whereas in others, for example polyesters [60], it does not.

The activation energies for the secondary γ- and β-relaxations are determined from this relaxation map for each of the two heating rates, and are given in Table 3. The average activation energy, *E*_a_, is 41.6 ± 0.4 kJ/mol for the γ-relaxation and 92.7 ± 6.3 kJ/mol for the β-relaxation. These values of the activation energy may be compared with those given in the literature for other hyperbranched polymers. Garcia Bernabé *et al.* have studied the relaxations in hyperbranched polyglycerol systems of different molecular weights (MW), denoted PG2.5, PG5 and PG10 in increasing order of MW [57,58], and report two secondary relaxations in the glassy state for each system: the γ-relaxation has activation energies of 38.6 ± 2 and 41 ± 2 kJ/mol for PG2.5, 45.4 ± 2 kJ/mol for PG5, and 35.7 ± 2 and 30 ± 6 kJ/mol for PG10, and is attributed to the terminal groups; the β-relaxation has higher activation energies, of 45.4 ± 2 kJ/mol and 81 ± 9 kJ/mol for PG2.5, 51.6 kJ/mol for PG5, and 49.0 ± 2 kJ/mol and 91 ± 5 kJ/mol for PG10, and is attributed to the motion of the linear hydroxyl groups. Kyritsis *et al.* also report two secondary relaxations for hyperbranched polyurethane systems [56]: the activation energy for the γ-relaxation, attributed to the end groups, is in the range 26–41 kJ/mol, while that for the β-relaxation, attributed to branched ends with polar groups, is in the range 60–72 kJ/mol. For hyperbranched polyamide systems, Hakme *et al.* [62] report values of 31 and 25 kJ/mol for the γ-relaxation and 55 and 50 kJ/mol for the β-relaxation, while Sangoro *et al.* [63] find values of 22 and 34 kJ/mol for the γ-relaxation, attributed to local methoxy groups, and 57 and 64 kJ/mol for the β-relaxation, attributed to the amino groups. For hyperbranched polyesters, Malmström *et al.* [59,60] report values of 96 ± 2 and 87 kJ/mol for the γ-relaxation of a five-generation polymer, attributed to the terminal hydroxyl groups, and very similar values of 96 ± 2 and 86 kJ/mol for the β-relaxation in the same system, attributed to the reorientation of the ester group, while Zhu *et al.* [61] find only the γ-relaxation in second and fifth generation systems, for which activation energies of approximately 133 and 100 kJ/mol may be calculated from their relaxation map. Finally, Turky *et al.* [84] report values of 22 to 35 kJ/mol for the γ-relaxation and 65 to 72 kJ/mol for the β-relaxation for various hyperbranched polyester amides.

Apart from the hyperbranched polyester systems, therefore, the appearance of two secondary relaxations is common, with the activation energy for the γ-relaxation being significantly smaller than that for the β-relaxation, as might be expected in consideration of the respective molecular motions involved; our results presented here also show this relationship. There is also a considerable variation in the activation energies for the different hyperbranched systems: for the γ-relaxation, it ranges from 22 to 45 kJ/mol, with much higher values being found for the polyester systems, while for the β-relaxation it ranges from 45 to 96 kJ/mol; our results are again in agreement with these observations, falling within this range of values.

For the α-relaxation, the parameters of the VFT equation:

ln*(f) = A − B/(T − T_0_)* (2)

are normally determined by a least squares fit of the VFT equation to the data, but for the results presented in Figure 7 there is insufficient range of temperature and frequency to obtain reliable values for these parameters. To demonstrate this, the best fit values for *A*, *B* and *T*_0_ are *A* = 26.4, *B* = 5028 K and *T*_0_ = 72 K for the heating rate of 0.5 K/min and *A* = 18.8, *B* = 2926 K and *T*_0_ = 108 K for the heating rate of 2 K/min, the values for *T*_0_ being considerably more than 100 K below *T*_g_, which does not seem reasonable. We comment further below on the fit of the VFT equation to experimental data in the context of other published results.

Instead of fitting the VFT equation, we calculate an “apparent” activation energy from the average slope of a best fit straight line to the data for the α-relaxation, such as those shown in Figure 7, which gives values of 78.1 kJ/mol and 63.3 kJ mol-1, for the heating rates of 0.5 and 2.0 K min^−1^, respectively, corresponding to the temperature range from 250 K to 300 K and 340 K, respectively. Differentiating Equation (2), one can also find this apparent activation energy, evaluated at an arbitrary temperature *T*, in terms of *B* and *T*_0_:

d ln*(f)/*d*(*1*/T) = −B/(*1 *− T_0_/T)^2^* (3)

Using the values for *B* and *T*_0_ above, and the average values for *T* of 275 and 295 K for the temperature ranges corresponding to heating rates of 0.5 and 2.0 K/min, respectively, these temperatures being between 60 and 80 K above the *T*_g_ determined by DSC, this gives apparent activation energies of 78.1 and 60.5 kJ/mol, in good agreement with the values above despite the unreasonably low values for *T*_0_.

These values for the apparent activation energy, and the method of analysing the α-relaxation in general, can be compared with reports in the literature for other hyperbranched polymers. For example, for their hyperbranched polyglycerol samples PG2.5 and PG10, Garcia Bernabé *et al.* [58] fit the VFT equation to their α-relaxation data, which consist of only 5 data points covering only about 20 K of temperature difference; this might be considered as an unreliable extrapolation to an asymptotic temperature *T*_0_, which is almost exactly 50 K below *T*_g_ for both samples. Nevertheless, using Equation (3) and their values of *B* and *T*_0_ one can calculate apparent activation energies of 249 and 309 kJ/mol for the two samples at their respective glass transition temperatures, as determined by DSC; at temperatures 60 to 80 K above *T*_g_ these values decrease to between 83.5 and 68.1 kJ/mol for PG2.5 and between 97.6 and 79.1 kJ/mol for PG10, similar to the values obtained above for Lupasol HBPEI.

Likewise, Sangoro *et al.* [63] investigated two novel hyperbranched polyamide amines, PAMAM1 and PAMAM2, and obtained parameters *B* and *T*_0_ by a best fit of the VFT equation to their α-relaxation data, covering less than 4 decades of the mobility of the charge carriers, over a temperature range of about 40 K well above *T*_g_; the values of *T*_0_ obtained are about 90 and 60 K, respectively, below the calorimetric *T*_g_ determined by DSC, which again might suggest a somewhat unreliable extrapolation. Using Equation (3) gives apparent activation energies at *T*_g_ of 183 and 324 kJ/mol, respectively, which reduce to 95.7 and 122 kJ/mol, respectively, at a temperature 70 K above *T*_g_.

On the other hand, Trahasch *et al.* adopt a different procedure for the analysis of the VFT fit to their data for dielectric relaxation in carbosilane dendrimers [51]. These authors recognise that the limited range of data available for the high frequency α-relaxation, covering less than 2 decades of frequency, would make the determination of *T*_0_ unreliable, and instead assume that *T*_0_ is 50 K below the calorimetric *T*_g_. The apparent activation energies in this high frequency region, calculated using Equation 3 with the fit parameter *B* and the assumed value for *T*_0_, and evaluated at a temperature 70 K above the respective *T*_g_’s for the three different samples studied, are 71.3, 64.6 and 70.6 kJ/mol. However, despite the fact that *T*_0_ is often found to be about 50 K below *T*_g_, as confirmed by the other literature results discussed above even though the *T*_0_ values are somewhat lower for the hyperbranched polyamide amines [63], the approach adopted here by Trahasch *et al.* cannot be considered to yield physically meaningful values for the other parameters *A* and *B*.

Similar inconsistencies in the analysis can be seen in the work of Turky *et al.* [84]. These authors investigate the dielectric relaxations in three hyperbranched polyester amides with hydroxyl (PEA-OH), phenyl (PEA-Ph), and stearate (PEA-St) terminal groups, and determine the VFT parameters from the temperature dependence of the rate of charge transport. The values of *B* (calculated as *DT*_0_ from their values of *D* and *T*_0_) are 989.8, 820.0 and 837.9 K, and of *T*_0_ are 202, 164 and 147 K, for PEA-OH, PEA-Ph and PEA-St, respectively. The apparent activation energies are found from Equation (3) as 314.2, 50.7 and 32.7 kJ/mol at their respective *T*_g_’s of 241, 259 and 273 K, and as 67.0, 27.1 and 24.8 kJ/mol at 70 K above their respective *T*_g_’s. These results are inconsistent, however, with the relaxation map shown in their Figure 3, where it can be seen that all three hyperbranched polymers display very similar behaviour with respect to charge transport, with apparent activation energies determined from the slope of these curves at *T*_g_ in the range from 160 to 180 kJ/mol, approximately. Furthermore, the values of *T*_0_ are 39, 95 and 126 K below their respective *T*_g_’s, which are again inconsistent with their appearance in the relaxation map, while the lowest value of *T*_0_, for PEA-St, implies an unreliably large extrapolation of their data. These VFT parameter values should therefore be treated with some caution in their interpretation.

We conclude from this discussion that our value, in the range of 60 to 80 kJ/mol, for the apparent activation energy for the α-relaxation in HBPEI at about 70 K above its *T*_g_ is consistent with values obtained for other hyperbranched systems. The same range of values is found for carbosilane dendrimers [51], while for hyperbranched polyglycerol [58] approximately the same range of values is found for the lower molecular weight sample PG2.5, with a slightly higher range of values being found for the higher molecular weight sample PG10, as might be expected. Similarly, the more complex molecular architecture of the hyperbranched polyamide amines results in a rather higher range of values [63], with that based on diethylenetriamine exhibiting a higher activation energy than that based on ethylene diamine, again as might be expected. On the other hand, the determination of the VFT parameters, and in particular *T*_0_, can be less reliable, and often requires what may be considered to be an unacceptably large extrapolation, particularly if the data are limited in their range of frequency and temperature.

#### 2.3.2. Epoxy

**Figure 8:**
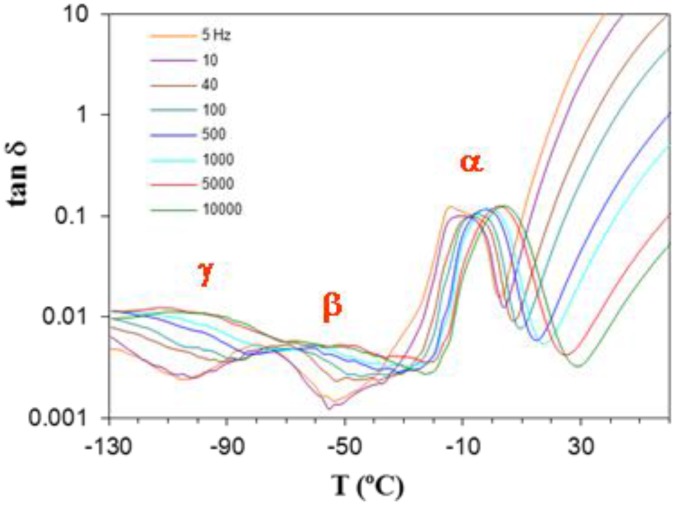
DRS relaxation spectrum for the neat epoxy resin at 2 K/min.

The dielectric spectrum of the neat epoxy resin is shown in Figure 8, where it can be seen that, similar to the HBPEI, it also shows three relaxations: a γ-relaxation, which is attributed to the movements of the epoxy groups; a β-relaxation, which is associated with the (-O-CH_2_-CH(OH)-CH_2_-) groups within the chain; and the α-relaxation, which is associated with the cooperative movement of the entire chain assembly. At high frequencies, there are essentially only the α- and γ-relaxations. With decreasing frequency, these two relaxations separate, and the β-relaxation begins to appear, between the α- and γ-relaxations, being less intense than the γ-relaxation. At temperatures above the α-relaxation, an increase in the signal is observed, corresponding to an ionic process.

**Figure 9:**
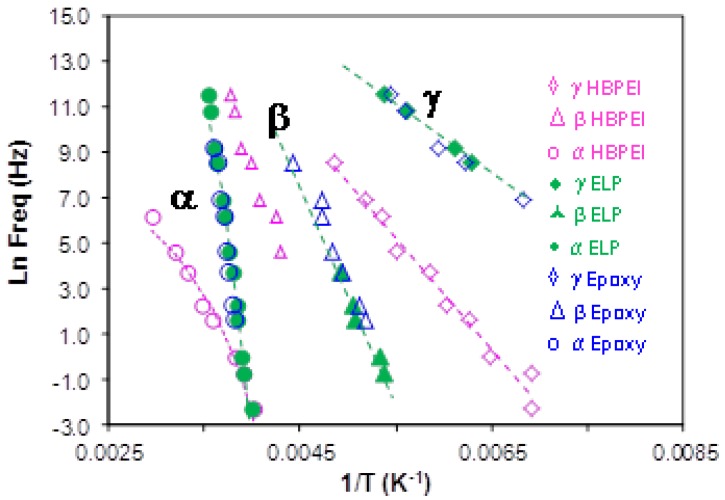
Relaxation map for the epoxy, HBPEI and ELP systems at 2 K/min. Blue open symbols, epoxy; pink open symbols, HBPEI; green symbols, ELP. Circles, α-relaxation; triangles, β-relaxation; rhombus, γ-relaxation.

The relaxation map for the neat epoxy resin is presented in Figure 9; also included in this Figure are the results for the HBPEI, discussed above and shown separately in Figure 7, and for the ELP mixture, to be considered below. Both of the secondary relaxations, β and γ, are considered to follow an Arrhenius temperature dependence, characterised by an activation energy, *E*_a_. The α-relaxation appears also to follow an Arrhenius behaviour, inasmuch as the data fit well to a straight line in Figure 9, and consequently it will be analysed as such, similar to the treatment of the HBPEI data above. In striking contrast with the HBPEI results, however, extrapolation of the line that describes the behaviour of the β-relaxation for the epoxy resin shows a possible merging with the α-relaxation at high frequencies. In this sense the β-relaxation seems to emerge from the α-relaxation and can be considered to be a JG type relaxation [83].

The activation energies for the secondary γ- and β-relaxations are given in Table 3. The activation energy value obtained for the γ-relaxation, 27.7 kJ/mol, is consistent with values given in the literature for a similar DGEBA epoxy resin: Casalini *et al.* [27] find 23.8 ± 0.8 kJ/mol, a value of 24.4 kJ/mol can be determined from the results of Capaccioli *et al.* [28], and Correzi *et al.* [29] report a value of 27.6 ± 0.5 kJ/mol. On the other hand, our value for the β-relaxation, 79.9 kJ/mol, is significantly higher than other values reported in the literature for DGEBA epoxy resins: Correzi *et al.* [29] find 47.6 ± 0.3 kJ/mol, Prevosto *et al.* [85] quote 47 kJ/mol, Serrano *et al.* [86] find 48.1 kJ/mol for an epoxy-diethylaniline mixture, and Mijovic *et al.* [87] obtain an even lower value of 39.2 kJ/mol. However, since the β-relaxation data for this epoxy and for the epoxy-HBPEI mixture, to be discussed below, are almost coincident, and the DMA analysis, also to be discussed below, likewise yields a similar value for the activation energy, we are confident of this result. Nevertheless, it appears strange that similar DGEBA resins should have significantly different values for the activation energies of the β-relaxation. An observation that may have a bearing on this is that in our DRS experiments the sample was heated at a constant rate, in this case at 2 K/min, from −130 °C to well above the β-relaxation, whereas in all the references quoted immediately above the procedure involved isothermal frequency scans after stabilising the sample at selected temperatures. This could be a plausible explanation if the β-relaxation were close enough to the calorimetric glass transition for structural relaxation effects to intervene; however, we do not believe this can have a significant effect in this case.

For the α-relaxation of the epoxy, there is insufficient range of frequency (5 Hz to 10 kHz) to make a reliable fit of the VFT equation to the data in Figure 9 for the heating rate of 2 K/min, and therefore a linear regression was made, which gives a value of 306 kJ/mol for the apparent activation energy over this range of frequencies, corresponding to a temperature range from 261.6 K to 277.3 K. This can be compared with the results of Fioretto and co-workers for a very similar DGEBA epoxy resin using DRS over a much wider frequency range [27,28,29]. These authors present relaxation maps, for which our data superpose on theirs very well in the range of frequencies used here, and determine the VFT parameters for a best fit, obtaining *B* = 720 K and *T*_0_ = 234.4 K, with a calorimetric *T*_g_ of 255 K [29]. Using Equation (3) we calculate an apparent activation energy at 269.5 K, the midpoint of our temperature range, of 352.9 kJ/mol, in good agreement with our own value.

It is interesting to note that the temperature *T*_0_ according to these authors lies only about 20 K below *T*_g_, considerably less than the approximately 50 K which is often assumed [51]. If we make use of the approximate relationship *x* = 1 − *T*_0_/*T*_g_ to calculate the non-linearity parameter, *x*, of the Tool-Narayanaswamy-Moynihan equation [88], we find *x* = 0.08; this is an incredibly low value. Although some values of *x* of this order have been reported previously from enthalpy relaxation studies [88,89], most notably a value of 0.10 for polyvinyl chloride [90], it is unusual; indeed, values of *x* significantly less than 0.20 were not even contemplated in the peak shift analysis of structural relaxation data [91]. Further insight can be gained by examining the values of *x* found from enthalpy relaxation studies in a series of epoxy resin systems. In order to investigate the effect of the degree of cure on the structural relaxation of epoxy resin, the non-linearity parameter was determined for a partially cured (70%) as well as a fully cured epoxy system [92,93], the value of *x* = 0.42 was found to be approximately the same in both systems. In subsequent work, the effect of cross-link length was investigated by using different curing agents [94], and it was found that *x* decreased as the network structure became more densely cross-linked. These observations seem to be contrary to the result obtained above where such a small value of *x* is associated with the unreacted epoxy resin. We believe that this is an aspect that deserves further study, and is the subject of another paper.

#### 2.3.3. ELP System

**Figure 10:**
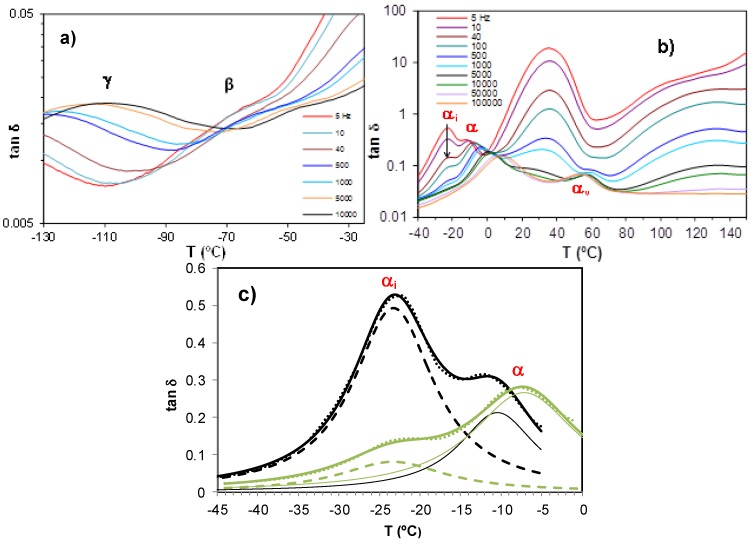
Dielectric relaxation spectrum for the ELP sample at heating rate of 0.5 K/min: (**a**) from −130 to −30 °C; (**b**) from −40 to 150 °C; (**c**) deconvolution of peaks at 5 Hz (black) and 40 Hz (green), showing experimental data (dotted lines), fit (thick lines), first peak (dashed lines) denoted α_i_ and second peak (thin lines) denoted α.

The dielectric spectrum of the uncured ELP system (epoxy and HBPEI mixture) has been obtained by a dynamic sweep from −130 °C to 150 °C at various heating rates. This temperature range comprises two distinct stages: the first, from −130 °C until the detection of the glass transition temperature, corresponds to the pre-cure stage; the second stage, until 150 °C, corresponds to the curing process of the sample. The results are shown in Figure 10 for a heating rate of 0.5 K/min. In the first stage, at temperatures below −25 °C and shown in Figure 10a, two secondary relaxations, the γ- and β-relaxations, are observed. In the second stage, shown in Figure 10b, the α-relaxation can be seen in the range of temperatures between −30 and 20 °C. This relaxation, at frequencies lower than 1 kHz, is overlapped with another peak which appears on its low temperature flank. The deconvolution of this composite peak, for the frequencies of 5 Hz and 40 Hz, can be seen in Figure 10c, where two peaks are clearly visible. A first peak, corresponding to an ionic process and occurring at a constant temperature of −23 °C, independent of frequency, is denoted α_i_, and a second peak, associated with a dipolar process and for which the temperature increases with increasing frequency, is denoted α and corresponds to the glass transition. These overlapping relaxations have only been detected in experiments at low heating rates and at low frequency. We believe that the relaxation peak denoted α_i_ in the uncured ELP system may be related to the glass transition of the HBPEI. The ELP system is phase separated, as has been seen in Figure 2, with the HBPEI as a minor component surrounded by epoxy resin. The DRS results indicate a much higher glass transition temperature for the HBPEI in the ELP mixture than in the neat state (−55 °C, see Table 2), and with a behaviour more similar to an ionic process than a dipolar one, both of which may be as a consequence of changes in the molecular mobility of the HBPEI in the two-phase morphology. This would explain why only a single glass transition was observed in the uncured ELP sample by DSC, which is not sensitive enough to detect these two overlapping transitions.

At temperatures above the α-relaxation glass transition temperature, the second stage corresponds to the curing reaction of the ELP system, seen clearly in Figure 10b. In this curing reaction there is a maximum in tan δ, occurring at a temperature which is not dependent on the frequency, and which is associated with the minimum viscosity of the system. The peak temperature is situated between 20 °C and 40 °C and is ionic in origin. With continued heating the curing reaction progresses, and at temperatures above 50 °C and for the higher frequencies another relaxation, denoted α_v_, becomes visible. The peak of this relaxation shifts to higher temperatures as the frequency decreases, is dipolar in origin, and is attributed to the vitrification of the system. This behaviour has also been observed in other curing systems [95].

**Figure 11:**
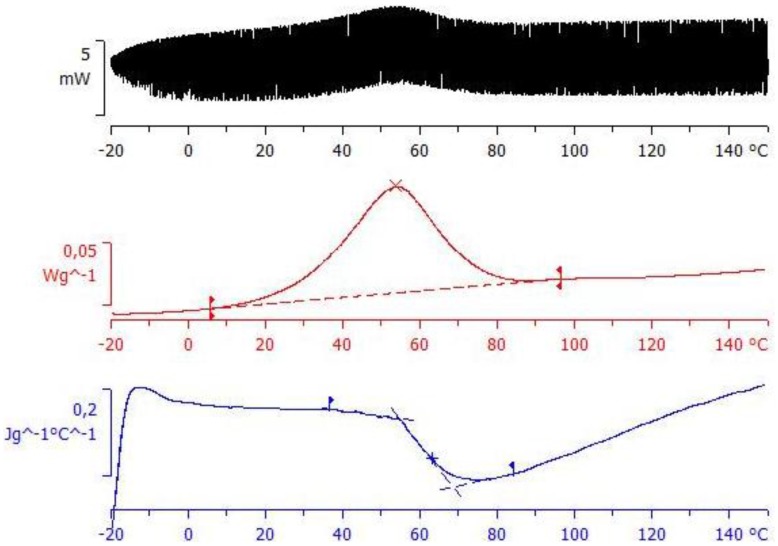
TOPEM thermogram of ELP at an underlying heating rate of 0.5 K/min: top diagram shows the stochastically modulated heat flow; middle diagram shows the total heat flow; bottom diagram shows the “quasi-static” specific heat capacity, *c*_p0_.

**Figure 12:**
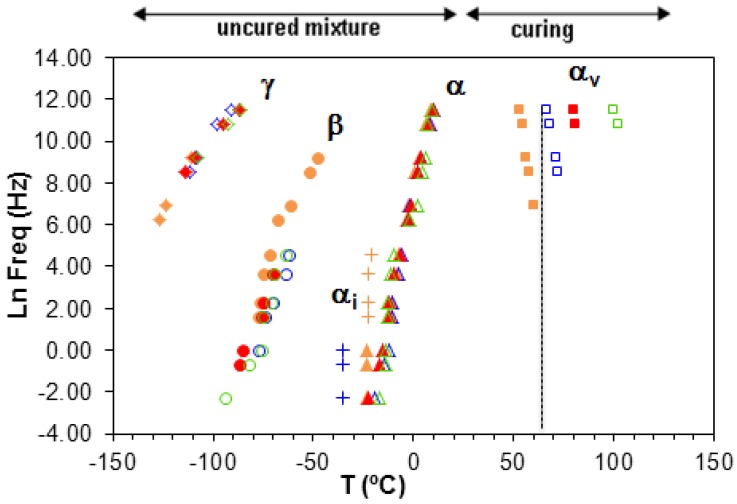
Temperatures of the relaxations peaks for the ELP sample at different heating rates: 0.5 K/min, orange filled symbols; 1 K/min, blue open symbols; 2 K/min, red filled symbols; 4 K/min, green open symbols. The vertical dashed line shows the vitrification temperature obtained by TOPEM at 0.5 K/min.

The vitrification process has also been verified by measurements made using TOPEM, a stochastic temperature modulated DSC technique [47,96], and the results are shown in Figure 11. The central diagram shows the overall heat flow, where a conventional exothermic curing reaction is clearly seen. The lower diagram, though, shows the reversing component, where a rather abrupt change occurs in the so-called “quasi-static” specific heat capacity, *c*_p0_, this change corresponding to the vitrification of the sample, as shown by Fraga *et al.* [47] and by Van Mele and coworkers [97]. The temperature at which vitrification takes place is measured as the midpoint of the sigmoidal change in *c*_p0_, and is found as 63.3 °C. This temperature corresponds well with the value found by means of dielectric measurements, shown in Figure 12 below, when extrapolated to the “quasi-static” frequency of approximately 4 mHz [98].

The temperatures of the relaxation peaks of the ELP sample at various heating rates are shown in Figure 12. The γ-, β- and α-relaxations show no significant dependence on the scan rate, whereas the α_v_-relaxation is significantly affected by the heating rate, occurring at higher temperatures the faster is the heating rate. This Figure also shows that this α_v_-relaxation is present over a wider range of frequencies the slower is the heating rate. This is exactly the dependence of the vitrification process on the heating rate that was shown earlier by TOPEM [99].

#### 2.3.4. Comparison of HBPEI, Epoxy and Uncured ELP

The comparison of the ELP sample with the pure components, epoxy and HBPEI, is made with respect to the relaxation map, already shown in Figure 9, from which the activation energies of the secondary relaxations and the apparent activation energy for the α-relaxation for the ELP sample are obtained and are given in Table 3. In Figure 9 it can be seen that the γ-relaxation for the ELP sample is coincident with that for the epoxy resin but quite distinct from that for the HBPEI, which also has a significantly different slope in this plot. Likewise for the β-relaxation, the ELP superposes on the epoxy but is quite distinct from the HBPEI, even though the slopes are approximately equal. Hence the values of the activation energies for the γ- and β-relaxations of the ELP sample are essentially the same as those for the epoxy, as can be seen in Table 3. This implies that the secondary relaxations in the ELP system reflect essentially those of the epoxy, with no significant contribution from the HBPEI.

**Figure 13:**
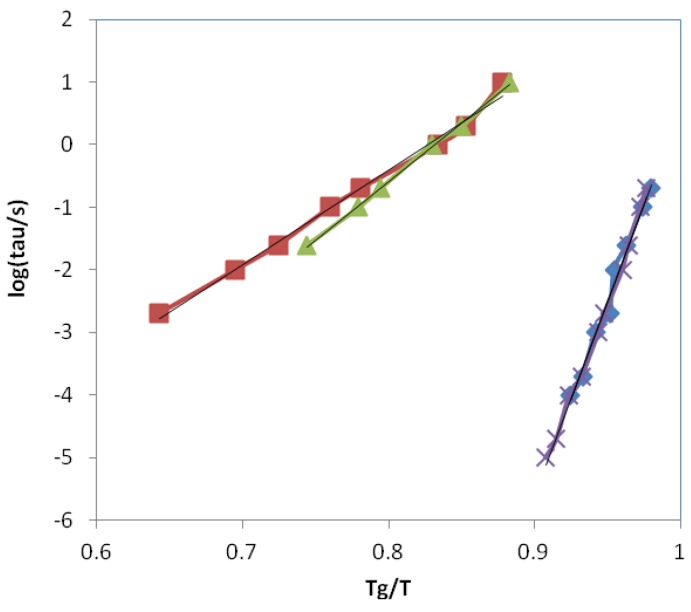
Fragility plot for epoxy, HBPEI and ELP samples: epoxy at 2 K/min, rhombus; HBPEI at 2 K/min, squares; HBPEI at 0.5 K/min, triangles; ELP at 0.5 K/min, crosses.

In respect of the α-relaxation, the ELP system is again coincident with that of the epoxy resin, and hence the apparent activation energy for the α-relaxation of the ELP system is essentially the same as that of the epoxy, again as can be seen in Table 3. The HBPEI relaxation behaviour is quite different, reflected in a significantly lower apparent activation energy, and showing considerably greater curvature than does either the epoxy or the ELP, though, as was shown above, the curvature and the limited frequency range are still insufficient for a reliable fit of the VFT equation to be made to the HBPEI data. If these data for the α-relaxation of the epoxy, HBPEI and ELP samples are plotted on a fragility plot of log(τ/s) *versus* *T*_g_/*T*, as shown in Figure 13, it can be seen that the extrapolation to the glass transition temperature *T*_g_, at which the average relaxation time τ is often considered to be 100 s, represented as the top right hand corner of Figure 13, presents an interesting difference. For the epoxy and ELP samples, for which the data are coincident, the relaxation time at *T*_g_ would be only 4 s, though some upward curvature characteristic of the VFT equation would increase this a little, and it is conceivable that the data could pass through log(τ/s) = 2 at *T*_g_. On the other hand, for the HBPEI at the two heating rates used the relaxation time at *T*_g_ would be much larger, between 400 and 1450 s, which would also be even larger if there were some upward curvature. The implication of this is that the HBPEI has a more restricted molecular mobility at *T*_g_ than is usually found.

Furthermore, Figure 13 shows clearly the quite different behaviours of the epoxy and ELP systems on the one hand and the HBPEI on the other. In fact, comparison with Angell’s *T*_g_-scaled fragility plot for the viscosity of glass-forming systems [100], or with the equivalent plot in which the average relaxation time is given [101], shows that these two systems are situated close to the extreme limits: fragile behaviour for the epoxy and uncured ELP samples, and strong behaviour for the HBPEI sample.

### 2.4. Dynamic Mechanical Analysis (DMA) Results

#### 2.4.1. Uncured Systems

**Figure 14:**
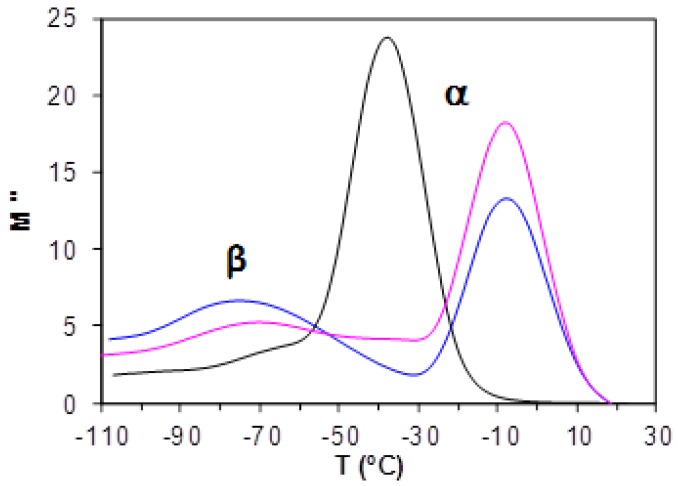
Loss modulus obtained by DMA at10 Hz and for a heating rate of 2 K/min for the three systems studied: epoxy, blue line; HBPEI, black line; uncured ELP, pink line.

The dynamic mechanical measurements of the pure components, epoxy and HBPEI, and of the uncured ELP system all exhibit a single secondary relaxation, the β-relaxation, in contrast to the two secondary relaxations observed by dielectric spectroscopy, together with the α-relaxation. This is illustrated in Figure 14 for the frequency of 10 Hz, where it can be seen that the spectrum for the ELP sample is similar to that for the epoxy, as was also observed in the dielectric measurements.

**Figure 15:**
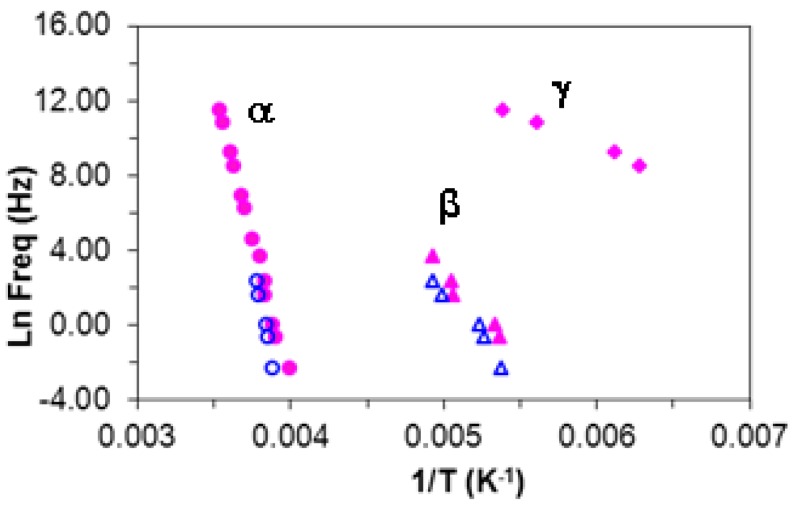
Comparative relaxation map for uncured ELP at 2 K/min obtained by two techniques: DRS, filled pink symbols; and DMA, open blue symbols.

From results such as those presented in Figure 14, and over a range of frequencies from 0.1 to 100 Hz, all at a heating rate of 2 K/min, a relaxation map for the ELP system can be constructed, and is shown in Figure 15 together with the dielectric results for comparison. It can be seen that both the β-relaxation and the α-relaxation are coincident when determined by the two different techniques. This is also reflected in the similar values found by the two techniques for the activation energies for the β- and α-relaxations, all of which are included in Table 3; the slightly higher value for the apparent activation energy for the α-relaxation determined by DMA could be because the frequency range is lower, and hence if there is some curvature according to a VFT relationship then the DMA technique would be sampling a region in which the apparent activation energy is indeed higher. Similar coincidence of dielectric and mechanical spectroscopy results has been observed previously in other epoxy systems [102].

#### 2.4.2. Cured ELP System

**Figure 16:**
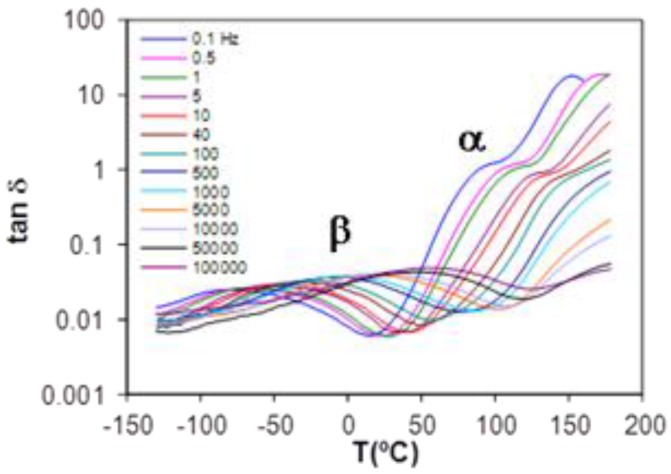
Dielectric spectrum of the cured ELP system at 2 K/min.

**Figure 17:**
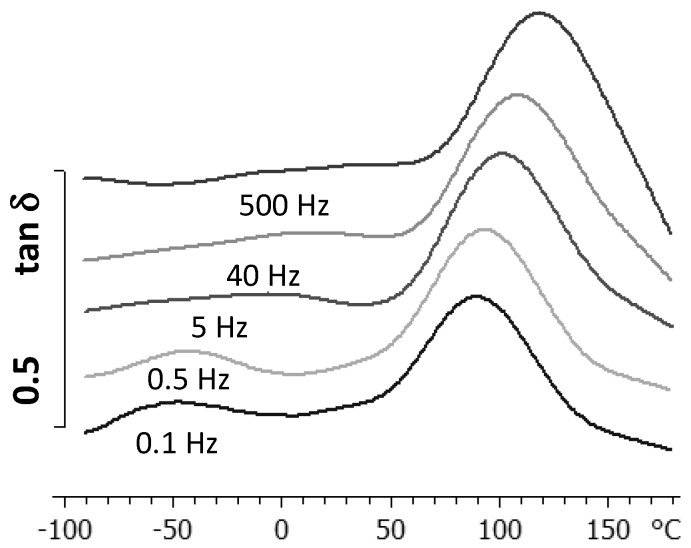
DMA spectrum of the cured ELP system at 2 K/min.

The study of the relaxations of the cured ELP system was performed using both DRS and DMA. The resulting dielectric spectrum, represented as tan δ, and the dynamic mechanical spectrum, represented also as tan δ, are shown in Figure 16 and Figure 17, respectively, each for a heating rate of 2 K/min. For both techniques, it can be seen that there is an α-relaxation at high temperature, well-defined in the DMA results but overlapping with conductivity effects in the DRS spectra, this α-relaxation being well separated from a secondary β-relaxation, which can clearly be seen over the whole range of frequencies used here, but there is no evidence of a γ-relaxation. The curing reaction in the ELP mixture consumes both the epoxide groups of the epoxy resin and the amine groups of the HBPEI (see Figure 4), to which were attributed the γ-relaxations in both of these pure systems, though it was shown above that the γ-relaxation in the uncured ELP system reflects only the contribution of the epoxy resin. Consequently, in the fully cured ELP system the γ-relaxation process does not appear.

A further DRS scan was made, at a heating rate of 5 K/min, on the same sample that had immediately previously been cured in the dielectric analyser at 0.5 K/min to give the results presented in Figure 10. From the frequency dependence of all these relaxation peaks, the activation energy of the β-relaxation and the apparent activation energy for the α-relaxation have been determined, and are given in Table 4. There are some surprising results.

Consider first the β-relaxation. The values of the activation energy obtained by DRS and DMA are very similar, as anticipated from their coincidence in the plot in Figure 15 for the uncured ELP system, and are also close to that reported by Tombari *et al.* for DGEBA cured with a diamine [41,103], namely 63.4 kJ/mol. However, the β-relaxation by DRS and DMA does not occur in the same temperature range. For example, from Figure 17 and for the frequencies of 0.1 and 0.5 Hz, for which the DMA peak is most clearly defined, it can be seen that the mechanical relaxation occurs in a temperature range about 20 K higher than that for the dielectric relaxation for the same frequencies. In contrast, while Okrasa *et al.* also find a displacement between the mechanical and dielectric relaxations in polyurethane networks cross-linked with an –OH terminated hyperbranched polyester [66], the mechanical relaxation in their systems occurs in a temperature range about 20 K lower than the dielectric relaxation. It appears that the dielectric and mechanical relaxations do not have the same origin, even though their activations energies are very similar.

Considering now the α-relaxation, it should first be noted that the dielectric spectrum appears to show two peaks; however, the apparent relaxation at the higher temperature is an effect of conductivity, and the α-relaxation really appears only as a shoulder. It is therefore difficult to assign a peak temperature accurately. Nevertheless, it is evident that the relaxation peak, for example at 0.1 Hz, occurs at a temperature much less than the glass transition temperature determined by DSC (~123 °C), whereas it would be expected to occur at a higher temperature. The same is true for the α-relaxation determined by DMA; furthermore, although the DMA and DRS α-relaxations occur in approximately the same temperature interval for the frequency of 0.1 Hz, their very different activation energies mean that their relaxation intervals diverge significantly at higher frequencies. Again, therefore, the relaxations identified by DMA and DRS appear to involve different mechanisms in this cured system, and both are different from that identified by DSC. This could be attributed to the inhomogeneous structure observed in the uncured epoxy-Lupasol mixture (Figure 2), which probably persists to a certain extent in the fully cured system. This is supported by similar observations made in the same epoxy systems cured with the same hyperbranched polymer but with different molecular weights [104], which will be reported elsewhere.

## 3. Materials and Methods

### 3.1. Materials

The epoxy resin used (DER 331, Dow Chemical Company) is a commercial diglycidyl ether of bisphenol-A (DGEBA), with an epoxy equivalent in the range 182–192 g/eq and a viscosity in the range 11,000–14,000 mPa s. The hyperbranched (HB) polymer used (Lupasol^®^ PR8515, BASF) is a commercial polyethyleneimine (PEI), with a molecular weight of 2000 g/mol and viscosity 12,000 mPa s. The chemical structure of the HBPEI is shown in Figure 1.

### 3.2. Preparation of the Samples

Before performing any of the experiments, the HBPEI Lupasol^®^ was previously pre-conditioned at 100 °C for 30 min to remove any moisture content in the sample. The preparation of the epoxy-HBPEI mixture, denoted ELP, with a composition of 83.4 wt % epoxy and 16.6 wt % HBPEI, was made by manually mixing the two pure components on a watch glass, followed by degassing in a vacuum chamber (Heraeus, model RVT360) for 10 min at room temperature and 100 Pa pressure. Immediately thereafter, proportions of this sample were taken in order to perform each of the corresponding experiments using the relevant thermal analysis techniques and equipment, and to observe the morphology under the optical microscope.

A cured plaque of the ELP mixture, of thickness 1.3 mm and lateral dimensions 25 mm square, appropriate for the dielectric analysis equipment, was prepared by isothermally curing in a Teflon mould, placed in a thermostatically controlled oven at 50 °C for 3 h, followed by a post cure at 140 °C for a further 3 h.

### 3.3. Experimental Techniques

A Leica DME polarising transmission optical microscope, equipped with a digital camera system, was used for observing the morphology of the ELP system.

Thermogravimetric analysis (TGA), which measures the weight loss of the sample during heating over a prescribed temperature range, was performed in a Mettler-Toledo TGA/DSC1 equipped with a sample robot and Huber cryostat (precision ± 0.1 °C). The TGA/DSC was calibrated using indium with a dry air flow of 200 mL/min and the experiments were performed with a dry nitrogen flow of 200 mL/min at heating rates of 2 K/min and 10 K/min over a temperature range from 40 °C to 600 °C.

The curing reactions were studied using a Mettler-Toledo DSC 821e differential scanning calorimeter (DSC) equipped with a sample robot and Haake EK90/MT intracooler. All DSC curing experiments were performed with a dry nitrogen gas flow of 50 mL/min. The data evaluation was performed with the STAR^e^ software. The DSC was calibrated for both heat flow and temperature using indium. A small sample of about 8–10 mg was weighed into an aluminium pan, sealed, and immediately inserted into the DSC furnace, whereupon the curing experiment was immediately started. Non-isothermal scans were made at rates of 0.5, 1, 2, 4 and 10 K/min over a temperature range from −65 °C to 200 °C, and were followed by a second scan at 10 K/min to determine the glass transition temperature, *T*_g∞_, of the fully cured sample.

A dielectric analyser DEA 2970 from TA Instruments was used to measure, in real time, the dielectric signals at different frequencies. Dielectric measurements were performed using a ceramic single-surface cell of dimensions 20 mm × 25 mm, based on a coplanar inter-digitated comb-like electrode design. The values of dielectric permittivity, ε′, and dielectric loss factor, ε″, were calculated from the resulting current and the induced phase angle shift, and the loss tangent tan δ was calculated as ε″/ε′. The interval of data sampling was 1 s per point. The sample was spread on the electrode surface, covering the entire inter-digitated area. The non-isothermal curing measurements were performed at temperatures from −130 °C to 200 °C in a nitrogen atmosphere with a gas flow of 500 mL/min. The heating rates were 0.5, 1, 2, and 4 K/min. The dielectric permittivity and the dielectric loss factor were measured at frequencies in the interval of 0.1 Hz to 100 kHz. Following each of the non-isothermal cure measurements, a second non-isothermal scan was made at a heating rate of 5 K/min from 30 °C to 200 °C.

Dielectric measurements for the ELP cured plaque were performed using a parallel plate sensor. In this case, non-isothermal measurements were performed at temperatures from −130 °C to 180 °C and the heating rate was 2 K/min.

Dynamic mechanical analysis (DMA) was carried out with DMA 861e (Mettler Toledo) in shear mode. The experiments for the pure components and for the uncured ELP mixture, all of which were viscous liquid samples, were performed using a special shear clamp over a temperature range from −130 °C to 25 °C, with a heating rate of 2 K/min. Measurements were made at intervals of frequency from 0.1 Hz to 100 Hz. The DMA experiment for the cured ELP, in the form of a disc of diameter 5.75 mm and thickness 1.3 mm, was carried out from −110 °C to 180 °C, with a heating rate of 2 K/min.

## 4. Conclusions

The dielectric and dynamic mechanical relaxations in an epoxy system cured with a hyperbranched polyethyleneimine, HBPEI, have been studied in experiments at constant heating rate. The pure components, epoxy and HBPEI, both display two secondary relaxations by DRS. For the HBPEI, the γ-relaxation is attributed to the –NH_2_ end groups and has an activation energy of 41 ± 2 kJ/mol, whereas the β-relaxation is attributed to the branches within the HBPEI structure and has a higher activation energy of 93 ± 7 kJ/mol; these activation energies are in agreement with other literature values. For the epoxy, the γ-relaxation is attributed to the epoxide groups and has an activation energy of 27 ± 2 kJ/mol whereas the β-relaxation has a much higher activation energy of 80 ± 4 kJ/mol; while the activation energy for the γ-relaxation is in agreement with other literature values, that for the β-relaxation is significantly greater, but corresponds closely to that found for the uncured mixture of epoxy and HBPEI. Besides these secondary relaxations, both the HBPEI and the epoxy display an α-relaxation which has been characterised by an apparent activation energy, *E*_app_, with values of 71 ± 8 kJ/mol and 306 ± 15 kJ/mol, respectively. Comparison of the α- and β-relaxations for these pure components shows that they have very different behaviours: whereas the β-relaxation for the epoxy has a Johari-Goldstein character, this is not the case for the HBPEI, for which the α- and β-relaxations appear completely separated.

The uncured mixture of epoxy and HBPEI also displays two secondary relaxations and a glass transition, which superpose almost exactly on the relaxation behaviour of the epoxy alone, implying the dominance of the epoxy in the uncured mixture. This is confirmed by DMA for the α- and β-relaxations; the γ-relaxation is not observed by DMA. In DRS at low heating rates and low frequencies, the α-relaxation splits into two, corresponding to the two phases observed by optical microscopy in the uncured mixture.

In the cured system, there is no γ-relaxation, as the end groups of the HBPEI and the epoxy groups have reacted. The β-relaxation is observed by both DRS and DMA, and has the same activation energy of 57 ± 4 kJ/mol, which is surprisingly significantly less than that for the uncured epoxy-HBPEI mixture. Although the activation energy is the same for these two techniques, though, the mechanical relaxation occurs in a temperature range about 20 K lower than that for the dielectric relaxation, indicative of different molecular processes involved. The α-relaxation by both DRS and DMA is found to occur at temperatures less than the glass transition temperature determined by DSC, namely 123 ± 3 °C. This is believed to be a consequence of the two-phase nature of the epoxy-HBPEI mixture.

## Figures and Tables

**Table 1:** TGA results for the different samples at the indicated heating rates.

Sample	Heating Rate (K/min)	*T*_5%_ (°C)	*T*_onset_ (°C)	*T*_DTGA_ (°C)
**ELP**	2	303.1	316.8	342.3
	10	305.1	346.9	373.6
**Epoxy**	2	289.8	315.6	351.5 and 410.3
	10	311.6	341.9	381.4 and 434.2
**HBPEI**	2	272.0	351.7	369.4
	10	301.2	379.1	398.8

**Table 2:** Differential scanning calorimetry (DSC) results for the HBPEI, epoxy and ELP samples.

Sample	Heating Rate (K/min)	*T*_g0_ (°C)	Δ*H* (J/g)	*T*_p_ (°C)	*T*_g∞_^a^ (°C)
HBPEI	2	−55.4			
	10	−54.0			
Epoxy	2	−17.2			
	10	−16.0			
ELP	0.5	−17.9	389	57.2	121.4
	1	−18.2	426	67.6	122.2
	2	−18.0	409	76.3	124.8
	4	−17.6	399	86.6	125.6
	10	−15.2	317	98.1	126.2
ELP Cured plaque	10				123.4

^a^ *T*_g∞_, determined at 10 K/min.

**Table 3:** Activation energies, *E*_a_, for the secondary relaxations, and “apparent” activation energies, *E*_app_, for the α-relaxation, for the HBPEI, epoxy and uncured ELP samples, obtained by DRS and dynamic mechanical analysis (DMA) at the different heating rates indicated.

Sample	Heating Rate (K/min)	DRS Relaxations			DMA Relaxations	
		γ	β	α	β	α
		*E*_a_ (kJ/mol)	*E*_a_ (kJ/mol)	*E*_app_ (kJ/mol)	*E*_a_ (kJ/mol)	*E*_app_ (kJ/mol)
HBPEI	0.5	42.0	86.4	78.1		
	2.0	41.0	98.9	63.3	99.0	209
Epoxy	2.0	27.7	79.9	306	89.0	358
ELP uncured	0.5	36.0	88.0	302		
	1.0	36.3	92.4	297		
	2.0	27.3	74.3	263	79.0	344
	4.0	26.5	71.6	273		

**Table 4:** Activation energies for the cured ELP system, obtained by DRS and DMA.

Sample	Heating Rate (K/min)	DRS Relaxations		DMA Relaxations	
		β	α	β	α
		*E*_a_ (kJ/mol)	*E*_app_ (kJ/mol)	*E*_a_(kJ/mol)	*E*_app_ (kJ/mol)
**cured ELP**	1	60.6	188		
	2	53.8	158	57.8	323
	5	-	172		
